# Early metabolic response using FDG PET/CT and molecular phenotypes of breast cancer treated with neoadjuvant chemotherapy

**DOI:** 10.1186/1471-2407-11-452

**Published:** 2011-10-20

**Authors:** Bhumsuk Keam, Seock-Ah Im, Youngil Koh, Sae-Won Han, Do-Youn Oh, Nariya Cho, Jee Hyun Kim, Wonshik Han, Keon Wook Kang, Woo Kyung Moon, Tae-You Kim, In Ae Park, Dong-Young Noh, June-Key Chung, Yung-Jue Bang

**Affiliations:** 1 Department of Internal Medicine, Seoul National University College of Medicine, Seoul, Korea; 2 Cancer Research Institute, Seoul National University College of Medicine, Seoul, Korea; 3 Department Nuclear Medicine, Seoul National University College of Medicine, Seoul, Korea; 4 Department of Radiology, Seoul National University College of Medicine, Seoul, Korea; 5 Department of Surgery, Seoul National University College of Medicine, Seoul, Korea; 6 Department of Pathology, Seoul National University College of Medicine, Seoul, Korea

**Keywords:** FDG PET, breast cancer, neoadjuvant chemotherapy, molecular phenotype

## Abstract

**Background:**

This study was aimed 1) to investigate the predictive value of FDG PET/CT (fluorine-18 fluorodeoxyglucose positron emission tomography/computed tomography) for histopathologic response and 2) to explore the results of FDG PET/CT by molecular phenotypes of breast cancer patients who received neoadjuvant chemotherapy.

**Methods:**

Seventy-eight stage II or III breast cancer patients who received neoadjuvant docetaxel/doxorubicin chemotherapy were enrolled in this study. FDG PET/CTs were acquired before chemotherapy and after the first cycle of chemotherapy for evaluating early metabolic response.

**Results:**

The mean pre- and post-chemotherapy standard uptake value (SUV) were 7.5 and 3.9, respectively. The early metabolic response provided by FDG PET/CT after one cycle of neoadjuvant chemotherapy was correlated with the histopathologic response after completion of neoadjuvant chemotherapy (*P *= 0.002). Sensitivity and negative predictive value were 85.7% and 95.1%, respectively. The estrogen receptor negative phenotype had a higher pre-chemotherapy SUV (8.6 *vs*. 6.4, *P = *0.047) and percent change in SUV (48% *vs*. 30%, *P = *0.038). In triple negative breast cancer (TNBC), the pre-chemotherapy SUV was higher than in non-TNBC (9.8 *vs*. 6.4, *P = *0.008).

**Conclusions:**

The early metabolic response using FDG PET/CT could have a predictive value for the assessment of histopathologic non-response of stage II/III breast cancer treated with neoadjuvant chemotherapy. Our findings suggest that the initial SUV and the decline in SUV differed based on the molecular phenotype.

**Trial Registration:**

ClinicalTrials.gov: NCT01396655

## Background

Breast cancer is second most common cancer in Korean women [1]. Neoadjuvant chemotherapy, also called preoperative chemotherapy, has emerged as the preferred initial component of therapy for patients diagnosed with locally advanced breast cancer. In the treatment of locally advanced breast cancer, multimodality approach, including neoadjuvant chemotherapy, surgery, and radiotherapy, is needed [2-4].

Over the last decade, the clinical value of fluorine-18 fluorodeoxyglucose positron emission tomography (FDG PET) in breast cancer has been widely studied [5-8]. However, a few studies have been conducted in stage II or III breast cancer patients receiving neoadjuvant chemotherapy, and the role of FDG PET remains inconclusive because of the small number of patients and heterogeneous regimens [9-13].

Furthermore, breast cancer is a heterogeneous disease with a demonstrated difference in prognosis based on molecular phenotypes. Many researchers have attempted to perform risk stratification and individualized treatment according to molecular phenotypes. We conducted a prospective phase II study of neoadjuvant docetaxel/doxorubicin chemotherapy in stage II or III breast cancer and recently reported the prognostic and predictive role of the molecular markers [14,15].

However, little is known how we consider the molecular phenotypes of breast cancer in order to interpret FDG PET findings [16]. This lead us to further investigate FDG PET in stage II or III breast cancer patients who received neoadjuvant chemotherapy, and FDG PET/CT was performed as a part of large clinical phase II study. The objectives of this study were 1) to investigate the predictive value of FDG PET/CT for histopathologic responses and 2) to explore the results of FDG PET/CT by molecular phenotypes of breast cancer.

## Methods

### Patients and treatment

**Figure 1:**
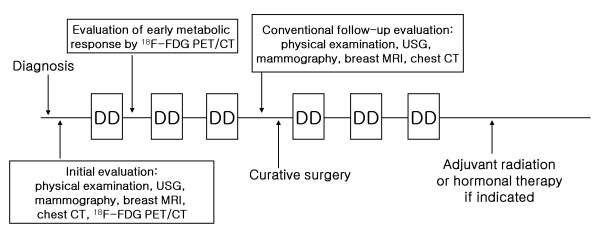
**Schematic flow of the neoadjuvant chemotherapy and response assessed by FDG PET/CT, breast MRI and chest CT**. Abbreviations: DD, docetaxel+doxorubicin; FDG PET, fluorine-18 fluorodeoxyglucose positron emission tomography; CT, computed tomography; MRI, magnetic resonance imaging; USG, ultrasonography.

Between July 2006 and September 2008, a total of 78 stage II or III breast cancer patients who received neoadjuvant docetaxel/doxorubicin chemotherapy were enrolled in this prospective study. The primary endpoint of this trial was evaluating pathologic complete response (pCR) rate, because pCR is repeatedly confirmed most relievable prognostic factor for neoadjuvant chemotherapy [17,18], and the best predictor of improved outcome and prolonged survival [3]. Secondary endpoints were evaluating survival, toxicity, predictive factors, and early metabolic response. The eligibility criteria were described in our prior reports [14,15]. In brief, the eligibility criteria were as follows: 1) pathologically-confirmed breast cancer by core needle biopsy, 2) initial clinical stage II or III, 3) objective measurable lesion, 4) ECOG performance 0~2, 5) previously untreated, and 6) adequate bone marrow, hepatic, cardiac, and renal functions. The patients received three cycles of neoadjuvant docetaxel/doxorubicin chemotherapy. The chemotherapeutic regimen consisted of docetaxel (75 mg/m^2^) and doxorubicin (50 mg/m^2^) by intravenous infusion every 3 weeks. After three cycles of neoadjuvant chemotherapy, the patients were re-evaluated for response and underwent curative surgery. Radiologic response was evaluated using breast magnetic resonance imaging (MRI) for the primary breast tumor and chest computed tomography (CT) for axillary, supraclavicular, internal mammary lymph nodes with RECIST criteria [19]. Both breast MRI and chest CT were performed in all the 78 patients. Subsequently, the patients received three more cycles of docetaxel and doxorubicin as an adjuvant chemotherapy, followed by hormonal or radiation therapy, if indicated [20]. Figure 1 shows the scheme for this study protocol. This regimen is known to be effective as neoadjuvant chemotherapy for stage II or III breast cancer [14,15,21]. We performed retrospective descriptive analysis for PET/CTs within this prospective study. This study protocol was conducted under the approval of the Institutional Review Board of Seoul National University Hospital (IRB approval number: 0510-506-159).

### Pathologic Assessment

The molecular markers that we have examined included estrogen receptor (ER), progesterone receptor (PR), human epidermal growth factor receptor 2 (HER2), and Ki-67. We performed immunohistochemistry (IHC) using tissues obtained before treatment and evaluated the association with clinical outcomes. IHC was performed as previously described [15,22]. The cut-off for ER and PR positivity was ≥ 10% positive tumor cells with nuclear staining. HER2 positivity was defined as either HER2 gene amplification by fluorescent *in situ *hybridization or scored as 3+ by IHC [23]. The molecular phenotypes of breast cancer were classified into luminal A, luminal B, HER2 and triple negative breast cancer (TNBC) [24]. TNBC was defined as ER(-), PR(-), and HER2(-). Luminal A phenotype was defined as ER(+) or PR(+) and HER2(-) tumor, and luminal B phenotype was defined as ER(+) or PR(+) and HER2(+). ER(-) and PR (-) and HER2+ tumor was classified as HER2 phenotype. Low expression of Ki-67 was defined as ≤5%. Histopathologic response was classified as proposed by Honkoop *et al*. [25] Specimens with no residual invasive carcinoma were classified as pCR. Residual ductal carcinoma *in situ *was included in the pCR category [26]. Specimens with only few scattered foci of microscopic residual invasive tumor were classified as minimal residual disease (MRD). Gross residual disease (GRD) comprised tumors showing macroscopic residual tumor or extensive residual tumor infiltration on microscopic examination. pCR and MRD were defined as histopathologic response and GRD as no histopathologic response.

### FDG PET/CT studies

Whole-body fluorine-18 FDG PET/CT scans were acquired before and after neoadjuvant chemotherapy for early metabolic response prediction. On the 15^th ^day of the first cycle of neoadjuvant chemotherapy, we obtained follow-up whole-body fluorine-18 FDG PET/CT scans. The CT scan protocol was as follows: tube voltage, 120 kV; current intensity, 50 mAs; scan time, 43.2 seconds; effective radiation dose, 4.8 mSv. FDG PET/CT was done using the same scanner (Gemini PET/CT system; Philips, Milpitas, CA, USA). Patients fasted for at least 6 hours before fluorine-18 FDG was injected intravenously. Patients were administered a weight-adjusted dosed of fluorine-18 FDG (5.18 MBq/kg), and images were acquired approximately 60 minutes (range: 50-75 minutes) after an intravenous injection of fluorine-18 FDG. Whole body emission scans were obtained for 2 minutes per bed position. PET/CT scanners automatically calculated the decay-corrected injected activity. We flushed the syringe and venous catheter three times, and residual activities in the syringe were less than 20 μCi and negligible. All patients were studied in the supine position with both arms raised above the head to pull their breast away.

Attenuation correction was based on the CT data, and PET data reconstruction was done by a 3-dimensional row action maximum likelihood algorithm. For quantitative assessment of tumor FDG uptake, region of interest were manually drawn on the slice with the highest radioactivity concentration of the primary breast tumor and in the adjacent slices.

The standard uptake values (SUVs) were calculated from the amount of FDG injected, body weight, and soft tissue uptake in the attenuation-corrected regional images as follows: SUV = (activity/unit volume)/(injected dose/body weight). Maximal SUV was defined as the SUV value on one pixel with the highest counts within the region of interest. The lesion chosen for analysis was the primary breast lesion with the highest SUV. The pre-treatment baseline maximal SUV of the tumor (pre-SUV) and the SUV of the tumor after the first cycle of chemotherapy (post-SUV) were compared with clinico-pathologic parameters. Calculation of the uptake index was as follows: ΔSUV% = 100 × (pre-SUV - post-SUV)/pre-SUV.

### Statistical analysis

This is a subproject of a phase II trial. The primary endpoint of the main project was pCR, and we designed this trial to test null hypothesis that the pCR rate was at most 10% against the alternative hypothesis that it was at least 20%. A sample size of 78 patients provided 80% power to test this hypothesis and α 0.05, using Simon's two stage minimax design [27]. The statistical consideration was performed according to the main project. The subproject has investigated the early metabolic change as primary endpoint. This was an exploratory analysis only, without an own sample size calculation for subproject. The primary objective of this subproject was to find a correlation between early metabolic response and pathological response. The association between histopathologic and metabolic response were assessed by a chi-square test. The cut-off of the SUV change for metabolic response was not prospectively defined. Receiver-operating characteristics (ROC) analysis was performed to determine a threshold for the prediction of metabolic response. The Mann-Whitney U test or Kruskal-Wallis test were used to compare SUVs according to different molecular phenotypes. All statistical tests were two-sided, with the level of significance established at *P *< 0.05. SPSS software (SPSS, Inc., Chicago, IL, USA) was used for all statistical analyses.

## Results

### Patients and results of treatment

The pretreatment characteristics are given in Table 1. Twelve patients (15.7%) were initially staged clinical stage II and the others (84.3%) were initial staged clinical stage III. The median follow-up duration was 18.7 months (range, 4.9-30.9 months). The tumor size was a median of 4.5 cm in the greatest dimension (range, 2.0-11.0 cm). At the end of follow-up, eight patients had developed recurrent disease, and one patient had died. The overall histopathologic response rate was 17.9%. Table 2 shows the results to neoadjuvant chemotherapy.

**Table 1:** Baseline characteristics of 78 patients

Characteristics	No. of Pt (%)
Median age (range)	45 (range 29-69)
Age < 50	56 (71.8)
Age ≥50	22 (28.2)
Performance status	
ECOG 0	23 (29.5)
ECOG 1	55 (70.5)
Pathologic characteristics	
Invasive ductal carcinoma	74 (94.9)
Others	4 (5.1)
Initial clinical stage	
IIA	1 (1.3)
IIB	11 (14.1)
IIIA	47 (60.3)
IIIB	13 (16.7)
IIIC	6 (7.7)
Inflammatory breast cancer	
Yes	3 (3.8)
No	75 (96.2)
Type of surgery	
Breast conserving	44 (56.4)
Mastectomy	34 (43.6)
Adjuvant hormonal therapy	
Yes	52 (66.7)
No	26 (33.3)
Adjuvant radiation therapy	
Yes	66 (84.6)
No	12 (15.4)

ECOG, Eastern Cooperative Oncology Group.

**Table 2:** Results to docetaxel plus doxorubicin neoadjuvant chemotherapy

Response	No. of Pts (%)
Histopathologic response	
Pathologic complete response	4 (5.1)
Minimal residual disease	10 (12.8)
Gross residual disease	64 (82.1)

### Changes in FDG PET/CT during neoadjuvant chemotherapy

The mean maximal SUVs ± standard deviation (SD) were as follows: pre-SUV = 7.5 ± 4.3 (range, 1.4~22.1), post-SUV = 3.9 ± 2.7 (range, 0~12), mean Δ SUV% = 40% (range, -260%~100%; SD = 46). The treatment response was assessed by breast MRI and chest CT for radiologic response and by FDG PET/CT for early metabolic response. Δ SUV% and decrease % of tumor diameter measured by RECIST criteria were well correlated (Pearson correlation coefficient = 0.267, *P *= 0.019).

**Figure 2:**
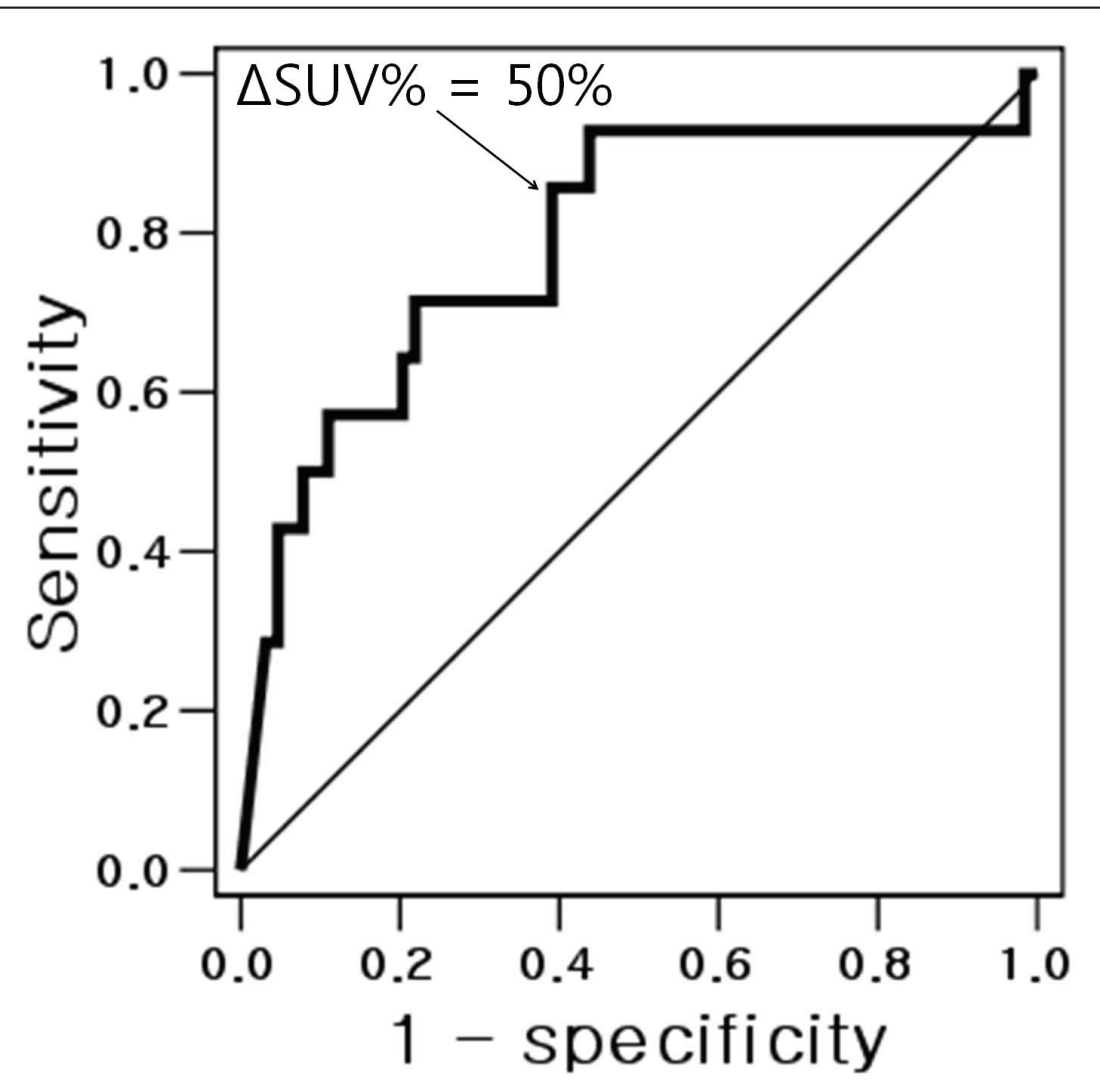
**Receiver-operating characteristics (ROC) analysis, determine optimal cut-off values of ΔSUV%; Area under the curve ROC was 0.79 (95% confidence interval 0.64-0.93)**.

ROC analysis were performed to determine optimal cut-off values of Δ SUV% to differentiate metabolic responder and non-responder, based on histopathologic response (Figure 2). The cut-off to achieve the highest positive predictive value was Δ SUV% of 50% (sensitivity of 85.7%, specificity of 60.9%). Among the all 78 patients, 37 patients (47.4%) achieved metabolic response, while 41 patients (52.6%) did not.

Δ SUV% was significantly greater in patients with histopathologic response than patients without histopathologic response (63% *vs*. 34%, *P *= 0.001, Table 3). Pre-SUVs were not different between histopathologic responder and non-responder, however post-SUVs were significantly differed, and this led to a difference of Δ SUV%. Histopathologic response was higher in early metabolic responders determined by FDG PET/CT after one cycle of neoadjuvant chemotherapy (*P *= 0.002) (Table 4). Among 14 histopathologic responder, 12 (85.7%) showed metabolic response. Importantly, 39 patients among the 41 metabolic non-responders (95.1%) were eventually revealed as histopathologic non-responder (Table 4).

**Table 3:** Correlation between response and SUV values.

Response	Pre-SUV	Post-SUV	ΔSUV%
Histopathologic response			
Responders	7.3 ± 4.2	2.2 ± 1.9	63 ± 48
Non-responders	7.6 ± 4.4	4.3 ± 2.7	34 ± 47
	*P *= 0.795	*P *= 0.004	*P *= 0.001

SUV, standard uptake value

*P*-value based on the Mann-Whitney U test, presuming to be non-parametric statistics

**Table 4:** Correlation between metabolic, and histopathologic response.

Response	Histopathologic responders *	Histopathologic non-responders	***P*-value**^**†**^
Metabolic responders			
Responders	12 (32.4%)	25 (67.6%)	0.002
Non-responders	2 (4.9%)	39 (95.1%)	

	For histopathologic responders	For histopathologic nonresponder	

Sensitivity	85.7% (12/14)	60.9% (39/64)	
Specificity	60.9% (39/64)	85.7% (12/14)	
Positive predictive value	32.4% (12/37)	95.1% (39/41)	
Negative predictive value	95.1% (39/41)	32.4% (12/37)	

* Pathologic complete response or minimal residual disease were regarded as histopathologic responders, Gross residual disease were regarded as histopathologic non-responders.

^† ^*P*-value based on the Fisher's exact test

### Changes in FDG PET/CT and histopathologic response according to molecular phenotype

**Figure 3:**
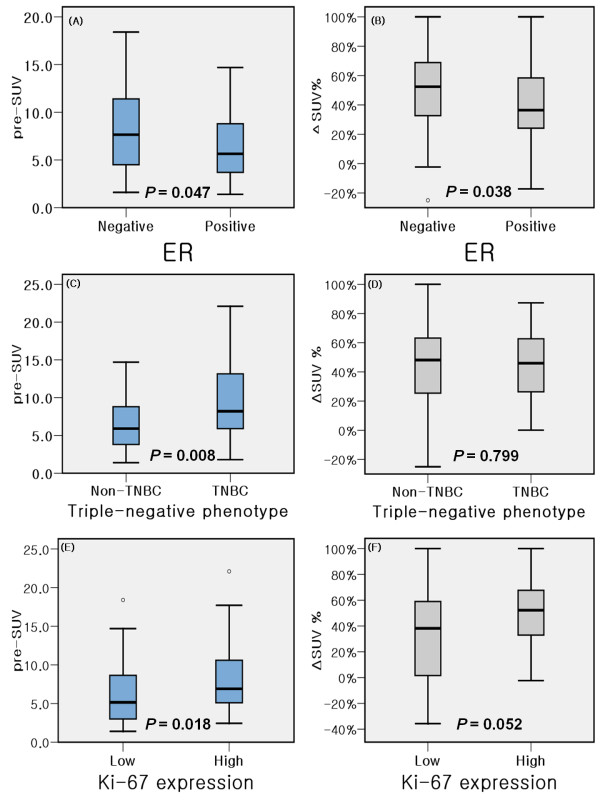
**Box flows comparing serial FDG PET/CT results according to molecular phenotypes based on immunohistochemistry**. ER negative phenotype showed higher pre-SUV (A) and Δ SUV% (B) than ER positive phenotype. In patients with high Ki-67 expression, a similar phenomenon was observed in that pre-SUV (C) and Δ SUV% (D) was higher than the low Ki-67 expression group. In triple negative breast cancer (TNBC), pre-SUV (E) was higher than non-TNBC; however, Δ SUV% (F) was not different.

The FDG PET/CT results were different according to the molecular phenotype of the breast cancer. The ER negative phenotype showed a higher pre-SUV (8.6 *vs*. 6.4, *P = *0.047) and Δ SUV% (48% *vs*. 30%, *P = *0.038) than ER positive phenotype. In patients with high Ki-67 expression, a similar phenomenon was observed in that the pre-SUV and Δ SUV% were higher than in the low Ki-67 expression group (8.5 *vs*. 6.2, *P = *0.018; 49% *vs*. 27%, *P = *0.052, respectively). In the TNBC group, the pre-chemotherapy SUV was higher than in the non-TNBC group (9.8 *vs*. 6.4, *P = *0.008); however, the Δ SUV% was not different (*P = *0.799). Table 5 and Figure 3 show the serial FDG PET/CT results according to molecular phenotypes. Histopathologic response rate was higher in ER negative patients than ER positive patients (27.5% *vs*. 7.9%, *P *= 0.037) and early metabolic response rate tend to higher in ER negative patients (57.5% *vs*. 36.8%, *P *= 0.068). In this context, histopathologic response rate was significantly different according to molecular phenotype (*P *= 0.018) and metabolic response tend to higher in HER2+ and TNBC subtypes than luminal type (66.7% *vs*. 50.0% *vs*. 40.0%, *P *= 0.255). Sensitivity and specificity of metabolic response for histopathologic response were higher in ER positive phenotype (100.0% and 68.6%, respectively) than those of ER negative phenotype (81.8% and 51.7%, respectively).

**Table 5:** SUV and changes in SUV based on molecular phenotypes

	No. of Pts	Pre-SUV *	Post-SUV *	ΔSUV% *	HPR (%)
ER					
Positive	38	6.4 ± 3.3	3.7 ± 2.1	30 ± 57	3 (7.9)
Negative	40	8.6 ± 4.9	4.1 ± 3.1	48 ± 37	11 (27.5)
		*P *= 0.047	*P *= 0.936	*P *= 0.038	*P *= 0.037^†^
PR					
Positive	30	6.5 ± 3.3	3.5 ± 2.4	36 ± 63	3 (10.0)
Negative	48	8.2 ± 4.8	4.2 ± 2.8	42 ± 37	11 (22.9)
		*P *= 0.187	*P *= 0.347	*P *= 0.837	*P *= 0.226^†^
HER2					
Positive	17	6.8 ± 3.2	3.2 ± 2.6	51 ± 36	5 (29.4%)
Negative	61	7.7 ± 4.6	4.1 ± 2.7	36 ± 51	9 (14.8%)
		*P *= 0.818	*P *= 0.247	*P *= 0.189	*P *= 0.164^†^
Triple negativity					
TNBC	26	9.8 ± 5.3	5.0 ± 3.0	42 ± 35	6 (23.1)
Non-TNBC	52	6.4 ± 3.2	3.4 ± 2.3	38 ± 54	8 (15.4)
		*P *= 0.008	*P *= 0.076	*P *= 0.799	*P *= 0.404^†^
Molecular phenotype					
Luminal A/B	40	6.3 ± 3.3	3.6 ± 2.2	33 ± 57	3 (7.5)
HER2	12	6.6 ± 3.2	2.8 ± 2.6	56 ± 39	5 (41.7)
TNBC	26	9.8 ± 5.3	5.0 ± 3.0	42 ± 35	6 (23.1)
		*P *= 0.016^‡^	*P *= 0.055^‡^	*P *= 0.150^‡^	*P *= 0.018^†^
Ki-67					
Low expression^**§**^	30	6.2 ± 4.1	3.6 ± 2.6	27 ± 65	4 (13.3)
High expression	41	8.5 ± 4.4	4.0 ± 2.8	49 ± 33	10 (24.4)
		*P *= 0.018	*P *= 0.541	*P *= 0.052	*P *= 0.367^†^

HPR, histopathologic responder; ER, estrogen receptor; PR, progesterone receptor; HER2, human epidermal growth factor receptor 2; TNBC, triple negative breast cancer.

* Data are presented as the mean ± standard deviation.

^† ^*P*-values are based on Chi square or Fisher's exact test.

^‡ ^*P*-values are based on Kruskal-Wallis test, otherwise *P*-values are based on Mann-Whitney U test, presuming to be non-parametric statistics.

**^§ ^**Low expression of Ki-67 was defined as ≤ 5%, and 7 patients were not available for Ki-67 results.

## Discussion

In the present study, we found that the early metabolic response using serial FDG PET/CT had a high predictive value for the histopathologic non-response of stage II/III breast cancer treated with neoadjuvant chemotherapy. Our study has demonstrated that the early metabolic response obtained on the 15^th ^day of first cycle of neoadjuvant chemotherapy may be useful in determining histopathologic non-responders with high negative predictive value of 95.1%, however, its value to identify histopathologic responders was low with positive predictive value of 32.4%. Different molecular phenotypes based on IHC reflect different metabolic properties and different histopathologic response rate.

Because current methods to monitoring tumor response (i.e., physical examination and conventional imaging modalities) have potential limitations [12], early metabolic responses by serial FDG PET/CT would be a useful complementary tool. Assessment of therapeutic response with serial FDG PET/CT scans was possible earlier than with any other method. Early evaluation of chemosensitivity *in vivo *using serial FDG PET/CT would help to identify non-responding patients, to avoid ineffective chemotherapy [28,29] and to decide the most appropriate therapeutic strategy. With given sensitivity of 85.7% and negative predictive value of 95.1%, metabolic non-responders identified by early serial FDG PET/CTs were likely to be histopathologic non-responders. In order to improve treatment outcome by risk stratification, the metabolic non-responder could be considered to have received another treatment strategy in earlier time course. It is necessary to design a future prospective randomized clinical trial comparing continuing standard cytotoxic chemotherapy alone versus incorporating new agents for metabolic non-responders to decide the clinical value of early determination of metabolic non-response.

Our study confirms previous reports [9,10,12,13,29-36] on the predictive value of changes in glucose metabolism after neoadjuvant chemotherapy in a prospective setting. Rousseau *et al*. [12] reported sensitivity of 61%, specificity of 96%, and negative predictive value of 68% when using FDG PET/CT after the first cycle of chemotherapy with a SUV cut-off of 60%. When comparing PET/CTs obtained baseline and 2nd cycles of chemotherapy, sensitivity and specificity were reported 73% and 63% with a cut-off of 45% [35], 93% and 75% with a cut-off of 50% [29], 77% and 80% with a cut-off of 40% [31]. The previous studies using FDG PET in neoadjuvant settings have indicated that measurements of SUV or changes in SUV during neoadjuvant chemotherapy allow prediction of histopathologic response as well as survival [9,10,12,13,29-37]. We used Δ SUV% because it was found to be the only independent predictive factor of histopathologic response in previous report [30]. Histopathologic response was higher in metabolic responder compared with metabolic non-responder (32.4% *vs*. 4.9%) although substantial portion of metabolic responder did not eventually get histopathologic response. Lower histopathologic response rate in our study was attributed to relatively short course of neoadjuvant chemotherapy in relatively advanced large tumors (median tumor size 4.5 cm). Importantly, 39 out of 41 metabolic non-responders did not get pathologic response which makes high negative predictive value (95.1%), and 85.7% of metabolic responders eventually showed pathologic response.

Bos *et al*. [16] reported that^18^FDG uptake in breast cancer was determined by the several biologic markers including glucose transporter-1, hexokinase I expression and mitotic activity index in breast cancer. Avril *et al*. [38] analyzed 56 operable breast cancer patients, and reported the positive correlation between baseline FDG uptake and tumor cell proliferation, which were similar with our results. In that report, hormone receptor negative breast cancer tended to have higher pre-SUV level than hormone receptor positive breast cancer, even though not reached statistical significance. However, little is currently known about the correlation between the SUV changes of FDG PET results and the meaning of molecular phenotype which is commonly used in the clinic to decide the treatment plan. In our study, breast cancer patients with high expression of Ki-67, which is regarded as highly proliferative and aggressive [39], showed a higher pre-SUV. ER negativity and high expression of Ki-67 was also associated with a high declining of the SUV. The pre-chemotherapy SUV of TNBC was higher than in non-TNBC, which was in line with other study [40].

Our study has some limitation. First, the median follow-up duration is relatively short with median follow-up duration of 18.7 months. As only few patients experienced relapses, we did not make an attempt to correlate the results of FDG PET/CT with survival. Second, the cut-off value of Δ SUV% should be validated in a larger study. Third, we did not obtain confirmatory statistical power for subgroup analysis.

However, our study was the first study exploring the metabolic uptake changes according to the clinically relevant molecular phenotype using serial FDG PET/CT before and after neoadjuvant chemotherapy. The patient population was highly homogenous in terms of stage and treated by same chemotherapy regimen and schedule. SUVs were obtained by the same PET/CT machine with the same protocol in a single institution. Metabolic lesions were well-matched because we used a combined PET/CT system.

## Conclusions

Despite some limitations, this study suggests that early metabolic response assessment with FDG PET/CT after the first cycle of neoadjuvant chemotherapy accuratly predicted histopathologic non-response and could be useful to identify patients undergoing ineffective or needing more aggressive chemotherapy. We also observed that the initial SUV and declining of the SUV differed based on molecular phenotype. Further prospective studies confirming the retrospectively calculated cut-off of 50%, and using longer duration of neoadjuvant chemotherapy with serial FDG PET/CT including early time point and just prior to definitive surgery are warranted.

## Abbreviations

FDG PET: fluorine-18 fluorodeoxyglucose positron emission tomography; CT: computed tomography; MRI: magnetic resonance imaging; ER: estrogen receptor; PR: progesterone receptor; HER2: human epidermal growth factor receptor 2; IHC: immunohistochemistry; TNBC: triple negative breast cancer; pCR: pathologic complete response; MRD: minimal residual disease; GRD: gross residual disease; SUV: standard uptake value; ROC: receiver-operating characteristics.

## Competing interests

The authors declare that they have no competing interests.

## Authors' contributions

Designing the concept of the study: SAI, BK. Provision of study patients and chemotherapy: SAI, SWH, DYO, JHK, TYK, YJB. Provision of study patients and surgery: WH, DYN. Pathologic examination and immunohistochemistry: IAP. Image analysis: NC, KWK, WKM, JKC. Data gathering, statistical analysis and interpretation: BK, YK. Manuscript writing: SAI, BK. All authors read and approved the final manuscript

## Pre-publication history

The pre-publication history for this paper can be accessed here:

http://www.biomedcentral.com/1471-2407/11/452/prepub
